# Supplementary material for: MicroRNAs of the miR-17~92 family maintain adipose tissue macrophage homeostasis by sustaining IL-10 expression
Source: eLife. 2020 Nov 5;9:e55676. doi: 10.7554/eLife.55676 (PMC7676864; doi:10.7554/eLife.55676)
Supplement: Supplementary file 1. [file elife-55676-supp1.docx]

**Supplementary file 1. Primers used in this study**

| Gene | Forward Primer (5’>3’) | Reverse Primer (5’>3’) |
| --- | --- | --- |
|  | Primer sequences for regular qPCR | |
| *Tnf*  *Il10*  *Yy1*  *Fos*  *Gapdh*  *Tnf-new*  *Il10-new* | GTCAGGTTGCCTCTGTCTCA  CTTTGCTATGGTGTCCTTTCA  GCCCTTTCAGTGCACATTCG  TCCTACTACCATTCCCCAGC  ATCAAGAAGGTGGTGAAGCA  TCCCCAAAGGGATGAGAAGT  GTAGAAGTGATGCCCCAGGC | TCAGGGAAGAGTCTGGAAAG  AAGACCCATGAGTTTCTTCAC  CTCCGGTATGGATTCGCACA  TGGCACTAGAGACGGACAGA  AGACAACCTGGTCCTCAGTGT  GCTCCTCCACTTGGTGGTT  GGGGAGAAATCGATGACAGC |
|  | Primer sequences for small RNA qPCR | |
| *miR-17*  *miR-20a*  *miR-20b*  *miR-106a*  *miR-106b*  *miR-93*  *miR-18a*  *miR-18b*  *miR-19a*  *miR-19b*  *miR-92*  *miR-25*  *miR-363*  *U6*  Universal reverse primer | CAAAGUGCUUACAGUGCAGGUAGU  UAAAGUGCUUAUAGUGCAGGUAG  CAAAGUGCUCAUAGUGCAGGUA  CAAAGUGCUAACAGUGCAGGUA  UAAAGUGCUGACAGUGCAGAU  CAAAGUGCUGUUCGUGCAGGUAG  UAAGGUGCAUCUAGUGCAGAUA  UAAGGUGCAUCUAGUGCAGUUA  UGUGCAAAUCUAUGCAAAACUGA  UGUGCAAAUCCAUGCAAAACUGA  UAUUGCACUUGUCCCGGCCUG  CAUUGCACUUGUCUCGGUCUGA  AAUUGCACGGUAUCCAUCUGUAA  TGGCCCCTGCGCAAGGATG | GCGAGCACAGAATTAATACGACTCAC |
