# Supplementary material for: MicroRNAs of the miR-17~92 family maintain adipose tissue macrophage homeostasis by sustaining IL-10 expression
Source: eLife. 2020 Nov 5;9:e55676. doi: 10.7554/eLife.55676 (PMC7676864; doi:10.7554/eLife.55676)
Supplement: Supplementary file 2. [file elife-55676-supp2.docx]

**Supplementary file 2.** ***Yy1* 3’UTR cloned fragments with miR-17 family miRNAs binding sites for luciferase reporter assays.**

(**1**) ***Yy1* 3’UTR fragments cloned for luciferase reporter assay**

AAAGAAGAGAGAAGACCTTCTCGACCCGGGAAGCCTCTTCAGGAGTGTGATTGGGAATAAATATGCCTCTCCTTTGTATATTATTTCTAGGAAGAATTTTAAAAATGAATCCTACACACTTAAGGGACATGTTTTGATAAAGTAGTAAAAATTTAAAAAATACTTTAATAAGATGACATTGCTAAGATGCTATATCTTGCTCTGTAATCTCGTTTCAAAAACAAGGTGTTTTTGTAAAGTGTGGTCCCAACAGGAGGACAATTCATGAACTTCGCATCAAAAGACAATTCTTTATACAACAGTGCTAAAAATGGGACTTCTTTTCACATTCTTATAAATATGAAGCTCACCTGTTGCTTACAATTTTTTTAATTTTGTATTTTCCAAGTGTGCATATTGTACACTTTTTGGGGATATGCTTAGTAATGCTGTGTGATTTTCTGGAGGTTGATAACTTTGCTTGCGGTAGATTTTCTTTAAAAGAATGGGCAGTTACATGCATACTTCAAAAGTATTTTTCCTGTACAAAAAAAAAGTTATATAGGTTTTGTTTGCTATCTTAATTTTGGTTGTATTCTTTGATGTTAACACATTTTGTATAATTGTATCGTATAGCTGTATTGAATCATGTAGAATCAAATATTAGATGTGATTTAATAGTGTTAATCAATTTAAACCCATTTTAGTCACTTTTTTTTCCCCAAAAAATACTGCCAGATGCTGATGTTCAGTGTAATTTCTTTGCCTGTTCAGTTACAGAAAGTGGTGCTCAGTTGTAGAATGTATTGTACCTTTTAACATCTGATGTGTACATCCGTGTAACAGAAAGGGCAACAATAAAATAGCGATCCTAAAGAAAGATTACGGCAGAAAGAGCTCTGTAAGCACAGCCTTATTTTCTTCTGTTGTCCAGAATACTTAGAATTCTTGAGCCTCCCAGAAATTGGAAGCAAATAAAGCAACTTGAGTTTCCTTTA

(**2**) **Predicted miRNA binding sites**

| **miRNA site** | **Position** | **Sequence** | **Mutated sequence** |
| --- | --- | --- | --- |
| miR-17 family miRNAs | 402-407 | CACTTT | ACTAGG |
| miR-17 family miRNAs | 688-693 | CACTTT | ACTAGG |
